# Enchondroma Detection from Hand Radiographs with an Interactive Deep Learning Segmentation Tool—A Feasibility Study

**DOI:** 10.3390/jcm12227129

**Published:** 2023-11-16

**Authors:** Turkka Tapio Anttila, Samuli Aspinen, Georgios Pierides, Ville Haapamäki, Minna Katariina Laitinen, Jorma Ryhänen

**Affiliations:** 1 Musculoskeletal and Plastic Surgery, Department of Hand Surgery, University of Helsinki and Helsinki University Hospital, 00029 Helsinki, Finland; 2 Department of Radiology, University of Helsinki and Helsinki University Hospital, 00029 Helsinki, Finland; 3 Musculoskeletal and Plastic Surgery, Department of Orthopedic Surgery, University of Helsinki and Helsinki University Hospital, 00029 Helsinki, Finland

**Keywords:** enchondroma, machine learning, deep learning, hand radiograph, segmentation, radiograph, benign tumor

## Abstract

Enchondromas are common benign bone tumors, usually presenting in the hand. They can cause symptoms such as swelling and pain but often go un-noticed. If the tumor expands, it can diminish the bone cortices and predispose the bone to fracture. Diagnosis is based on clinical investigation and radiographic imaging. Despite their typical appearance on radiographs, they can primarily be misdiagnosed or go totally unrecognized in the acute trauma setting. Earlier applications of deep learning models to image classification and pattern recognition suggest that this technique may also be utilized in detecting enchondroma in hand radiographs. We trained a deep learning model with 414 enchondroma radiographs to detect enchondroma from hand radiographs. A separate test set of 131 radiographs (47% with an enchondroma) was used to assess the performance of the trained deep learning model. Enchondroma annotation by three clinical experts served as our ground truth in assessing the deep learning model’s performance. Our deep learning model detected 56 enchondromas from the 62 enchondroma radiographs. The area under receiver operator curve was 0.95. The F1 score for area statistical overlapping was 69.5%. Our deep learning model may be a useful tool for radiograph screening and raising suspicion of enchondroma.

## 1. Introduction

Benign bone tumors encompass a diverse spectrum of neoplasms, and their classification hinges on the specific matrix they generate, whether it be bone, cartilage, fibrous tissue, fat, or blood vessels [1]. The two most common primary cartilage-forming tumor subtypes are osteochondroma, which manifests as a sessile outward-growing tumor from the bone, and enchondroma, characterized by its growth within the bone itself [1]. While osteochondromas and giant cell tumors hold the distinction of being the most prevalent benign bone tumors throughout the body, enchondromas exhibit their highest incidence in the hand [1,2,3]. Enchondromas have a characteristic appearance in radiographs, where the tumor area looks hollow and the bone cortices are thin, but also an atypical or more un-noticeable presentation is possible especially in larger bones outside the hand [1,2]. Although many enchondromas are discovered incidentally, the most common symptoms are swelling, pain, and deformity, and the diminished bone cortices, also referred as endosteal scalloping, predispose the bone to fracture. The most typical lesion location in the hand is in the proximal phalanges. Diagnosis is based on clinical examination and radiographic imaging [2]. Despite their typical appearance on radiographs, enchondromas can be misdiagnosed or may go totally unrecognized when a fracture is present [4]. Diagnostic errors, especially in emergency rooms, are common and may cause patient harm [5]. Malignant transformation from primary enchondroma to chondrosarcoma is possible but extremely rare [6]. The differential diagnosis between enchondroma and chondrosarcoma grade I/atypical cartilaginous tumor (ACT) remains a challenge due to histological similarities and overlapping of histological criteria.

The rise in computational power over recent decades, coupled with advances in artificial intelligence applications, particularly in image classification and object detection and segmentation, has sparked significant interest across various research fields [7]. Machine learning (ML) has already been successfully used in a broad spectrum of medical settings in ophthalmology, pathology, dermatology, and radiology [8]. Deep convolutional neural networks, a subgroup of ML, have found significant application in the domains of image classification and segmentation. In our study’s respective field, hand radiographs were previously evaluated by Eng et al., who found that their deep learning (DL) model improved skeletal age assessment performed by radiologists [9]. Several studies have assessed rheumatoid arthritis and joint destruction detection in hand radiographs with promising results [10,11,12]. Üreten et al. also included osteoarthritis differential diagnostics in their study. When looking at previous bone tumor research with ML techniques from conventional radiographs, Yu et al. developed a DL model to differentiate primary bone tumors as benign, intermediate, or malignant with comparable results to subspecialists and superior results compared to junior radiologists [13]. In a study conducted by Park et al., the researchers found that their DL model, developed to classify hip radiographs in three groups (benign, malignant, or no tumor), performed superior to subspecialists and concluded that their DL model may reduce misdiagnosis [14]. Do et al. successfully trained a Multi-Level Seg-Unet model for the detection and classification of knee bone tumors, achieving remarkably high levels of accuracy [15]. 

ML techniques have also been used with computed tomography (CT) and magnetic resonance imaging (MRI) images for bone tumor detection, segmentation, and classification. In a study by Eweje et al., preoperative MRI images were used to train a DL model for tumor classification (benign vs. malignant) and received comparable results to an expert committee. In the context of differentiating between chondrosarcoma and enchondroma, various studies have explored the utility of CT and MRI images, with and without the application of radiomics techniques [16]. Gitto et al. conducted a study that investigated the differential diagnosis of atypical cartilaginous tumors and chondrosarcoma using radiomics in both CT and MRI modalities, achieving good to high levels of accuracy [17,18]. Additionally, another study focused on utilizing MRI radiomics for distinguishing chondrosarcoma from enchondroma. In this research, pathology served as the gold standard for comparison, and different models were evaluated, all of which demonstrated strong performance in this diagnostic task [19]. These imaging modalities are usually used for treatment planning after suspicion has been raised with a traditional radiograph and is thus not available for diagnostic aid. 

Still, no studies on ML-based hand enchondroma detection have been performed. A large amount of preceding data is typically required for meaningful use of ML. Image augmentation, e.g., scaling and rotating, and choosing appropriate deep learning (DL) techniques, such as segmentation, can be attempted to address this drawback [20]. We believe that by using the segmentation approach it is possible to develop a feasible DL model even within settings where data are inherently scarce. 

We developed a DL model to detect enchondromas from hand radiographs and compared the DL model’s results on the test set with those of three clinical experts (two hand surgeons and one musculoskeletal radiologist). We hypothesized that the developed DL model would be capable of detecting enchondromas with comparable accuracy to clinical experts.

## 2. Materials and Methods

The research committee of Helsinki University Hospital (HUS/379/2020/4) approved the study and waived the need for informed consent. This study was completed in accordance with the principles of the Declaration of Helsinki.

A retrospective cohort of 500 enchondroma radiographs from 82 patients treated in our tertiary hospital between 1 January 2003 and 31 December 2019 was formed. We searched the hospital’s bone tumor meeting records, the Nomesco (Nordic Medico-Statistical Committee) procedural classification codes (NDR*, NDQ*, NDK*, and ZZH*), and the Helsinki University Hospital pathological archives using histopathological diagnosis for “enchondroma” or “chondroma” [21]. Patients’ medical records and radiographs were reviewed, and available and suitable radiographs were included. Postoperative radiographs were excluded. Available radiographs of the contralateral hand without an enchondroma were also collected. 

The radiographic finding was verified as an enchondroma via postoperative histopathology (95%) or through expert consensus (5% in cases where the decision was made in a bone tumor meeting). Five cases of clinically typical lesions showed no growth tendency in follow-up radiographs, and thus, there was no need for operative treatment.

**Figure 1:**
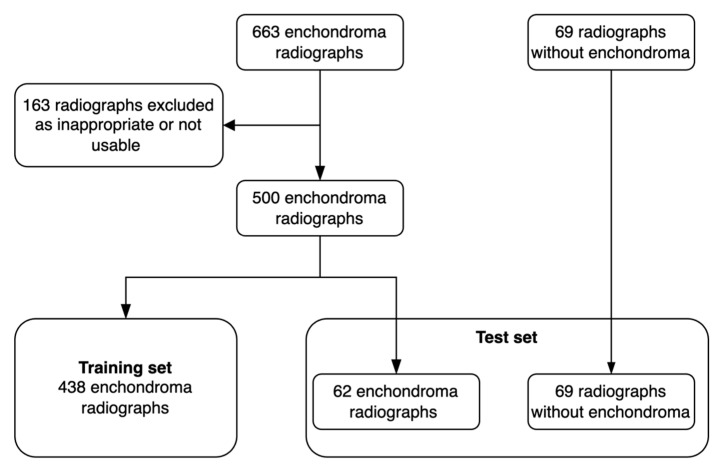
Flowchart of the radiograph acquirement process. Training set = radiographs used for DL model training; test set = radiographs used for comparing the DL model’s results against clinical experts.

The radiographs were acquired from the hospital’s Picture Archiving and Communications System (PACS), pseudonymized, and converted to png format by a data analyst. Radiographs with summation between the enchondroma and other bony structures were excluded. We used Aiforia Create version 5.3, a third-party cloud-based interactive segmentation software that uses pixel-level segmentations for the DL algorithm’s training [22]. The software allows the clinician to bypass the typical need for programming and enables the user to choose and fine-tune the DL algorithm’s features to suit the task. The software visualizes the DL model’s training and development status such that the additional segmentations can be used for the most difficult features of the image that the algorithm has not yet learned. The enchondroma radiographs were divided into a training set and a test set patient-wise. Radiographs without enchondroma were added to the test set. The radiograph division process is outlined in Figure 1 and distribution of radiographs between different projections and presence of fracture is shown in Table 1. 

The DL model was first trained with a layer to differentiate bone tissue from soft tissue and the background; for this layer, the algorithm complexity “Complex” was chosen. 

After the DL model’s bone tissue detection rate started to coincide with that of the annotator, training was continued with a child layer to detect enchondroma from healthy bone tissue. All training set enchondroma radiographs were segmented to maximize the training data for enchondroma detection. All segmentations were manually drawn with the software’s standard annotation instruments by one hand surgeon (T.A.) on the radiographs using a mouse. For the annotator (T.A.), the assessment of the performance of the DL model was possible by reanalyzing and visualizing the training set radiographs. Additional segmentations were made when considered beneficial. 

The algorithm was trained for enchondroma detection using the following parameters and standards augmentations, where radiographs are automatically augmented in the training process by adjusting scale (−1 to 1.01), aspect ratio (1), maximum shear (1), luminance (−1 to 1), contrast (−1 to 1.01), maximum white balance change (1), noise (0), JPEG compression quality (40 to 60), blur (1 pixel), JPEG compression percentage (0.5), blur percentage (0.5), and rotation angle (−180 to 180). Image flipping was enabled, and the initial training rate was set to 1 with minimal batch size of 20. The algorithm complexity “extra complex” was chosen for this task. Aiforia Create was running AI engine version 2 when the training was completed. The neural network was initially configured to train for 17,000 iterations, but it terminated at the 16,021st iteration since the last 500 iterations did not lead to any additional improvement in the model’s probability for detecting enchondroma. “Iterations without progress” is another modifiable parameter of Aiforia Create that enables the DL model developer to govern the training process. 

The test set radiographs were also annotated by one hand surgeon (T.A.) with knowledge of whether an enchondroma was present in the radiograph. To evaluate the DL model’s performance from a segmentation point of view, rather than pure binary assessment, we used F1 score, also known as Dice coefficient [23]. F1 score is a statistical measure utilized to quantify the degree of overlap between two sets of data; in our particular scenario, this was between the assessments made by the DL models and the annotator.

The test set radiographs and the DL model’s drawings were analyzed by a hand surgeon (T.A.). To produce a binary classifier, the result was converted to a binary result (correct/incorrect), and the algorithm’s parameter “gain” was changed gradually between 0.01 and 10.0 in the analysis. This produces changes in the network’s sensitivity and specificity, which can be plotted in the receiver operator characteristic (ROC) curve. The DL model provides a confidence percentage for every region of interest that it predicts. We used a confidence threshold of 70% for a region to be considered positive. The area under the curve was calculated with 95% confidence interval bootstrapping 10^3^ samples. Confidence intervals for sensitivity, specificity, and accuracy are “exact” Clopper–Pearson confidence intervals. The ROC curves were calculated using R-software (version 4.2.3.) [24].

To compare the DL model’s performance against clinical experts, two hand surgeons and one musculoskeletal radiologist analyzed the test set radiographs separately using the annotation tools from the development environment. Interrater agreement of the experts was assessed with Cohen’s kappa, and performance was evaluated as the mean of the three assessments, as suggested by McHugh [25].

## 3. Results

**Figure 2:**
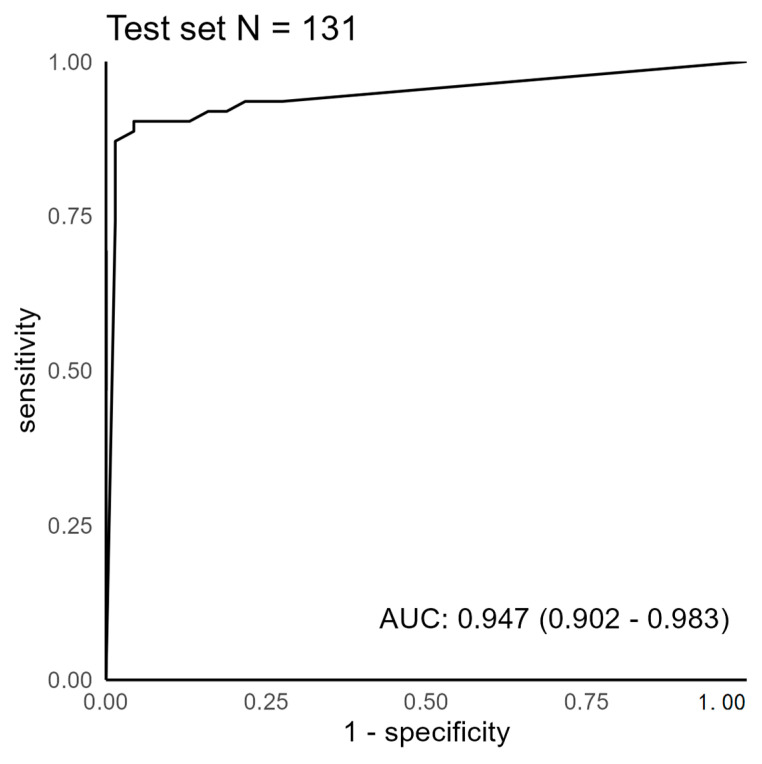
Receiver operator characteristic curve for enchondroma detection. AUC = area under the curve.

Our DL model detected 56 of 62 enchondromas in the test set. The model falsely predicted three areas as positive in 69 radiographs without enchondroma. In six radiographs, the DL model predicted an enchondroma in healthy bone tissue in adjunct to a correct enchondroma finding. In the training set, an F1 score of 0.82 was acquired. For the DL model’s predictions that overlapped with the annotator’s (T.A.) drawings in the test set, an F1 score of 0.70 was obtained. The area under the receiver operator characteristic curve was 0.947, as shown in Figure 2. On comparing the different results gained, the operating point of the DL model was determined to be 0.7. 

**Figure 3:**
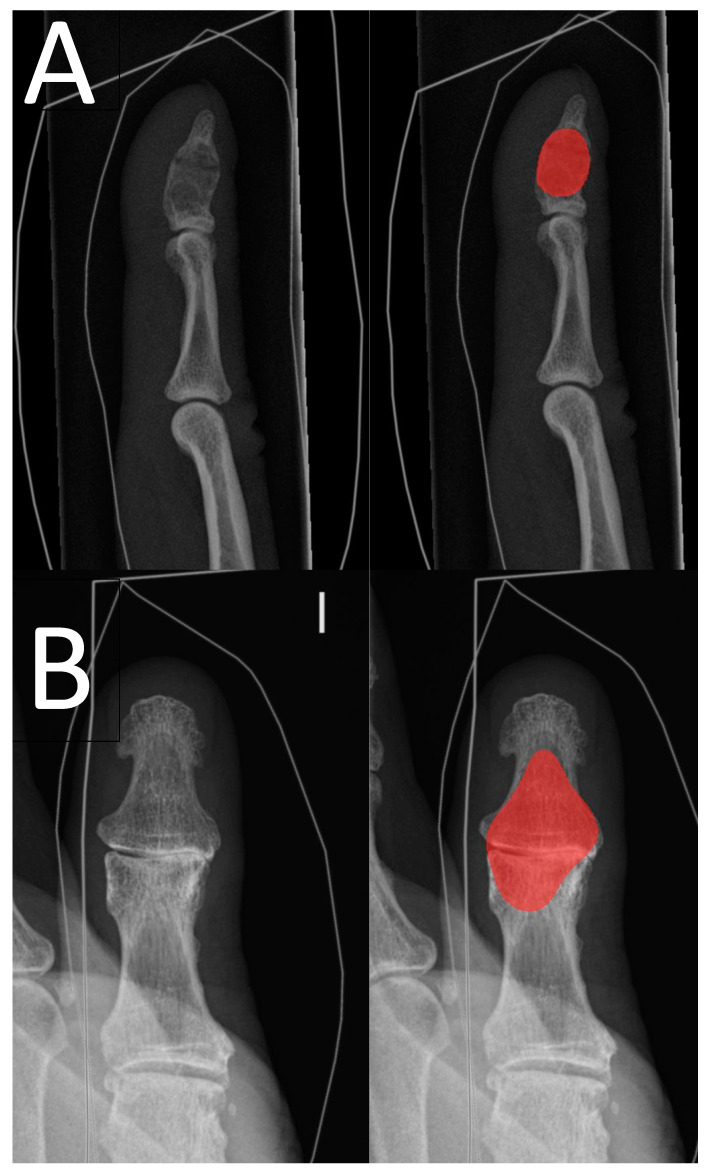
Examples of the DL model’s predictions. Image pair (**A**) shows a distal phalange true-positive prediction (red area) of an enchondroma with a pathological horizontal fracture, and image pair (**B**) shows a false-positive prediction (red area) of thumb interphalangeal joint area with osteoarthrosis.

The inter-rater reliability of the clinical experts was good, with a value of 0.96. The results are presented in more detail in Table 2. Clinical expert 2 drew an enchondroma in healthy bone tissue in adjunct to the correct drawing in one enchondroma radiograph. Table 3 shows the confusion matrix for the DL model’s enchondroma detection. In Figure 3, correct and incorrect predictions of the DL model are shown.

## 4. Discussion

Hand radiographs serve as a pivotal diagnostic tool for various injuries and symptoms. Although enchondroma is common in the hands, its primary diagnosis can be challenging. Automated radiograph screening with a precise and tireless DL model would improve and expedite diagnostic accuracy and quality.

Our results show that automated enchondroma detection is feasible and may facilitate screening of radiographs for enchondromas both as a “pre-reader” and as a clinical aid for emergency room doctors not routinely treating hand problems or viewing radiographs. 

Our clinical experts showed good results, and the inter-rater reliability was good. One may ponder the need for our DL model as clinical experts perform so well. In acute settings, hand trauma patients are often treated by general practitioners, and an expert (hand surgeon, orthopedic surgeon, or radiologist) is not always immediately available. With our model in clinical use, it could highlight the potential enchondroma and flag the image for consultation. Detecting enchondromas can also be challenging for radiologists. Additional training material with confounding changes, such as cysts caused by osteoarthrosis, is needed for the model to be widely utilizable. With additional training using radiographs and, in unusual cases, MRIs to serve as ground truth, we believe that a DL model may outperform radiologists and clinicians in the future. Hopefully, DL models will also be able to detect enchondroma-associated fractures and to assess fracture alignment in the future, aiding in optimal treatment decisions based on features of the enchondroma and its location in the bone.

This is the first research paper to study automated DL-based enchondroma detection from hand radiographs to our knowledge. Other DL models that have been developed based on hand radiographs include those for rheumatoid arthritis detection, joint subluxation, and skeletal age assessment [9,10,11,12]. A few studies have also investigated primary bone tumor detection and malignancy classification [13,14], but not in the hand. A previous study conducted by Moldovan et al. delved into segmentation techniques, employing them to identify different fracture fragments of dislocated proximal tibial fractures and to facilitate the automated alignment of the fragments during 3D planning with computed tomography images [26]. The versatile nature of segmentation enables its application in various contexts, including our specific use case, where we harnessed segmentation to distinguish the tumor area from the surrounding healthy bone tissue. In our view, to ensure the dependable evaluation of acute radiographs by a DL model, it is imperative to take into account even less common conditions like enchondromas. Otherwise, the DL model assessment may give clinicians a false sense of security and raise the threshold for consultation, thus leading to patient harm due to delayed or missing treatment.

Our training set radiographs encompassed all typical projections of hand radiographs, also with similar distribution in the test set. The reduced number of lateral projections is primarily attributable to our exclusion of radiographs with an overlap of adjacent bony structures covering the enchondroma.

In our training set data, there was a fracture present in almost one third of the radiographs. This shows that even though enchondromas are usually discovered incidentally, acute health care visits can also be due to pathological fractures. Fractured enchondromas may also be over-represented in our data as some small incidentally detected lesions may be controlled only in the primary healthcare setting without specialist consultations. The lower occurrence of fractures in the test set radiographs is due to our control radiographs without enchondromas. 

The same interactive cloud-based artificial intelligence environment utilized in our research has previously demonstrated its usability, especially in the analytics of histopathological slides [27,28]. However, our study represents a pioneering application of this software for radiograph analysis. We have several strengths in our study that point to the DL model’s robustness and reliability. 

Firstly, in the majority of cases in our study, the diagnosis was histologically confirmed, reinforcing the accuracy of our findings. Also, the extensive experience of our clinical experts, who annotated the test set radiographs, indicate that the DL model performs at a high level. The meticulous pixel-level annotations used for DL model training may have enabled the development of an accurate DL model with limited training data. Furthermore, the visualization of the DL model’s predictions on the training set aided in the annotation process to find the most challenging characteristics of the image for the DL model. The radiographs were taken with several different radiograph devices in regular use during the time, which enhances the robustness of the DL model.

However, our study has certain limitations that need to be acknowledged and addressed. The relatively small number training radiographs may impact the model’s generalizability. Overfitting is also a concern in ML research, particularly when the algorithm’s performance is finely tuned, especially when dealing with limited training data. Another limitation in our study is the absence of a distinct validation set for assessing the performance of the DL model between training iterations, potentially leading to the risk of overfitting. The nature of our binary outcome within the Aiforia platform made the inclusion of a separate validation set unfeasible. The disparity between the F1 scores of the training and test sets may be attributed overfitting. In reality, the magnitude of this difference is not as pronounced as the raw numbers suggest. This is primarily due to Aiforia’s inability to omit predictions with confidence levels between 50% and 70% from the calculation of the F1 score. In the training set, these predictions between 50 and 70% were few, but in the test a more significant number of predictions were excluded because they did not meet the 70% confidence threshold. In ROC curve generation and other metrics, this was considered and only predictions with confidence values over 70% were included. This discovery emphasizes the need for additional validation, both in our institution and with a new separate cohort, and underscores the imperative requirement for external validation to assess the generalizability of the DL model. Another limitation is the possibility of near-invisible enchondromas that may have been missed in both the training and the test set radiographs, potentially affecting the model’s performance. Also, there is the possibility of falsely outlined enchondromas in the training set radiographs, stemming from the challenge of two-dimensional images depicting the three-dimensional structure of enchondromas. 

Evaluating the efficacy of the DL model necessitates a comprehensive analysis encompassing its influence on patient care, its impact on diagnosis time and the prevention of misdiagnosis, as well as an appraisal of its cost-effectiveness. It is imperative to ascertain these to fully evaluate the model’s real-world utility. Further DL model development plans from radiographs could be the differential diagnosis of enchondroma and chondrosarcoma from plain radiographs, as previously performed from MRI and CT with ML, texture analysis, and radiomics [17,18,19]. Due to the challenges and constraints associated with the histological diagnosis of peripheral chondrosarcoma and enchondroma, the DL model could offer an additional valuable assessment to assist in determining the most suitable treatment for these patients. Further training with additional enchondroma-associated pathological fractures may also allow for the development of a DL model for pathological fracture detection and the evaluation of fracture alignment. With further fine-tuning of the DL model, it may be possible to give suggestions in the future.

## 5. Conclusions

In conclusion, hand radiographs are a crucial diagnostic tool for assessing various injuries and symptoms. However, diagnosing enchondroma, a relatively common condition in the hands, can pose a challenge. Our study demonstrates the feasibility of automated enchondroma detection, which has the potential to serve as a valuable “pre-reader” for radiographs and as a clinical aid for emergency room physicians who may not routinely specialize in hand-related issues or hand radiograph interpretation. Future DL model development plans may involve differential diagnosis of enchondroma and chondrosarcoma from plain radiographs. Given the challenges of histological diagnosis for these conditions, the DL model could offer valuable support in determining treatment strategies. Further training with additional enchondroma-associated pathological fractures could enable the development of a DL model for fracture detection and evaluation of fracture alignment. With continued refinement, the DL model may provide valuable treatment suggestions in the future. To assess the real-world utility of the DL model, it is crucial to evaluate its impact on patient care, diagnosis time, prevention of misdiagnosis, and cost-effectiveness.

## Figures and Tables

**Table 1:** Showing the distribution of radiographs between different projections and occurrence of fracture.

	Anteroposterior View	Oblique View	Lateral View	Fracture in Enchondroma	Total Amount
Training set	207 (47%)	157 (36%)	74 (17%)	141 (32%)	438
Test set	58 (44%)	47 (36%)	26 (20%)	29 (22%)	131

**Table 2:** Results with 95% confidence intervals of the DL model compared with results of clinical experts.

	DL Model	Clinical Expert 1	Clinical Expert 2	Clinical Expert 3
Sensitivity	0.90 (0.80–0.96)	1.00 (0.94–1.00)	1.00 (0.94–1.00)	1.00 (0.94–1.00)
Specificity	0.96 (0.88–0.99)	0.95 (0.87–0.99)	0.99 (0.93–1.00)	1.00 (0.95–1.00)
Accuracy	0.93 (0.87–0.97)	0.97 (0.93–0.99)	0.99 (0.96–1.00)	1.00 (0.97–1.00)
AUC	0.95 (0.90–0.98)			

**Table 3:** Confusion matrix for enchondroma detection.

Radiographs		Actual		
Predicted		Tumor	Normal	
	Tumor	56	3	59
	Normal	6	66	72
		62	69	131

## Data Availability

Due to legislation, the radiographic data cannot be shared.
